# Potential Immune Biomarker Candidates and Immune Subtypes of Lung Adenocarcinoma for Developing mRNA Vaccines

**DOI:** 10.3389/fimmu.2021.755401

**Published:** 2021-11-30

**Authors:** Yang Wang, Huaicheng Tan, Ting Yu, Xiaoxuan Chen, Fangqi Jing, Huashan Shi

**Affiliations:** ^1^ Department of Biotherapy, Cancer Center, West China Hospital, Sichuan University, Chengdu, China; ^2^ Laboratory of Aging Research and Cancer Drug Target, State Key Laboratory of Biotherapy, National Clinical Research Center for Geriatrics, West China Hospital, Sichuan University, Chengdu, China; ^3^ Department of Pathology and Laboratory of Pathology, State Key Laboratory of Biotherapy, West China Hospital, West China School of Medicine, Sichuan University, Chengdu, China; ^4^ State Key Laboratory of Oral Diseases, National Clinical Research Center for Oral Diseases, West China Hospital of Stomatology, Sichuan University, Chengdu, China; ^5^ Department of Radiotherapy, Cancer Center and State Key Laboratory of Biotherapy, West China Hospital, Sichuan University, Chengdu, China

**Keywords:** mRNA vaccine, lung adenocarcinoma, immune subtype, tumor mutation burden, immune landscape, immunogenic cell death, immune checkpoint, immune biomarker

## Abstract

mRNA vaccines against cancer have advantages in safety, improved therapeutic efficacy, and large-scale production. Therefore, our purpose is to identify immune biomarkers and to analyze immune status for developing mRNA vaccines and selecting appropriate patients for vaccination. We downloaded clinical information and RNA-seq data of 494 LUAD patients from TCGA. LUAD mutational information was hierarchically clustered by *NMF* package (Version 0.23.0). *DeconstructSigs* package (Version 1.8.0) and NMF consistency clustering were used to identify mutation signatures. *Maftools* package (Version 2.6.05) was used to select LUAD-related immune biomarkers. TIMER was used to discuss the correlation between genetic mutations and cellular components. Unsupervised clustering Pam method was used to identify LUAD immune subtypes. Log-rank test and univariate/multivariate cox regression were used to predict the prognosis of immune subtypes. Dimensionality reduction analysis was dedicated to the description of LUAD immune landscape. LUAD patients are classified into four signatures: T >C, APOBEC mutation, age, and tobacco. Then, GPRIN1, MYRF, PLXNB2, SLC9A4, TRIM29, UBA6, and XDH are potential LUAD-related immune biomarker candidates to activate the immune response. Next, we clustered five LUAD-related immune subtypes (IS1–IS5) by prognostic prediction. IS3 showed prolonged survival. The reliability of our five immune subtypes was validated by Thorsson’s results. IS2 and IS4 patients had high tumor mutation burden and large number of somatic mutations. Besides, we identified that immune subtypes of cold immunity (patients with IS2 and IS4) are ideal mRNA vaccination recipients. Finally, LUAD immune landscape revealed immune cells and prognostic conditions, which provides important information to select patients for vaccination. GPRIN1, MYRF, PLXNB2, SLC9A4, TRIM29, UBA6, and XDH are potential LUAD-related immune biomarker candidates to activate the immune response. Patients with IS2 and IS4 might potentially be immunization-sensitive patients for vaccination.

## Introduction

Lung adenocarcinoma (LUAD) is a leading cause of death worldwide with over 1 million deaths annually and accounts for approximately 40% of lung cancers (1, 2). LUAD progresses quickly with the existence of micro-metastatic foci, high tumor recurrence rate, and increased tumor metastasis rate. When the cancerous lesions are small, the blood vessels and lymph nodes might be invaded. Numerous patients have no opportunity of surgery in stages IIIB and IV (3). Therefore, a systematic treatment is essential for cancer control. Another obstacle is low chemotherapy/radiotherapy sensitivity and drug resistance of targeted therapy resulting from a second gene mutation. Cancer immunotherapy is a breakthrough in the 21st century. Programmed cell death-1 (PD-1), programmed cell death-1 ligand (PD-L1), and cytotoxic T-lymphocyte-associated antigen 4 (CTLA-4) therapies are effective in some patients with melanoma or non-small-cell lung cancer (4–6). Therapeutic vaccines activate the T cell to recognize cancerous neoantigens, initiating an immune response for individualized vaccination (7). Therefore, LUAD requires combined therapy and we should classify the LUAD population with different molecular characteristics (8).

During the epidemic of Corona Virus Disease 2019 (COVID-19), mRNA vaccines have evolved as a novel therapeutic method to fight against cancer and infectious diseases (7, 9). The superiorities in safety, efficacy, and the industrial production enables mRNA vaccines to be a promising therapeutic tool for individualized treatment (10). Immunologically, the immunogenicity of mRNAs is reduced by modifying nucleotides chemically, optimizing mRNA with GC-rich sequence and adding poly (A) tails (11–13). Biologically, mRNA exists in the cytosolic plasma, instead of integrating into genomic DNA in the nucleus. The stability of mRNA in the cytosolic plasma is realized by sequence optimization and carriers (lipid, polymer, peptide, particle, and cationic nanoemulsion) (10, 14–16). A good illustration of mRNA-vaccine efficacy is that RNActive (CureVac AG) vaccine platform activated T cell immunity and exerted good tolerability and immunogenicity in a Phase Ib study for patients with non-small cell lung cancer (17). Moreover, the mRNA-related cost/benefit ratio are low, because of large-scale production and rapid development. If we hope developing individualization-oriented therapy for LUAD, mRNA vaccines can be designed to encode pathological antigens that elicited effective immune responses. There are two ongoing clinical trials (NCT03908671 and NCT02688686) that applied suppressor of cytokine signaling (SOCS) 1-, MUC1-, and survivin-encoding mRNAs to treat non-small cell lung cancer.

However, other kinds of tumor vaccines (tumor cell vaccines, DNA vaccines, peptide vaccines, and dendritic cell vaccines) have some limitations. First, the development of tumor cell vaccines is refrained by poor clinical efficacy (18). Second, vaccinated DNA would enter the nucleus to participate in the transformation of genomic DNA (19, 20). Third, peptide vaccines involve MHC-restricted short peptides which means that these peptides are restricted to selected antigens and epitopes (21). Finally, for dendritic cell vaccines, not all dendritic cells are matured. The production of dendritic cells is time-consuming (22). A common disadvantage of these four vaccines is that peptide genetic analysis for individualized treatment would delay treatment and that the analysis is impossible for inoperable tumors (23).

Currently, no LUAD immune biomarkers have been identified, owing to tumor heterogeneity and different tumor microenvironment. It is essential to select LUAD population for vaccination. Therefore, our purpose is to identify LUAD immune biomarkers for developing mRNA vaccines and to validate immune status (immune subtypes and landscape) for determining the vaccination population. The procedure of our study is illustrated in **Supplementary Figure 1**.

## Materials and Methods

### Data Collection and Pre-Processing

We downloaded clinical information and RNA-seq data of 494 LUAD patients from the Cancer Genome Atlas (TCGA) (https://www.cancer.gov/tcga) by the Genomic Data Commons (GDC) platform. Samples lacking clinical information and genes with zero Fregments Per Kilobase per Million (FPKM) in 50% of samples were excluded. For further analysis, data standardization was realized by FPKM-Transcripts Per Million (TPM) transition. Furthermore, 2,212 immune-related genes were collected from gene sets, including antigen processing and presentation, BCR signaling pathway, chemokines and chemokine receptors, cytokines and cytokine receptors, interleukins, natural killer cell cytotoxicity, TCR signaling pathway, TNF family members, TGF-β family members, and other immune related gene sets.

### The Analysis of LUAD Mutation Signature and LUAD-Related Immune Biomarkers

Alexandrov et al. described that the mutational processes of human cancer can be downloaded from the website (http://cancer.sanger.ac.uk/cosmic/signatures) (24). We accessed TCGAbiolinks to download 567 LUAD specimens in TCGA database. Their mutational information was hierarchically clustered by using a R package (non-negative Matrix Factorization, NMF) which was based on 96 trinucleotide mutation spectra (25). The optimal R value identified four major mutation features of LUAD (**Supplementary Figure 3**). A R package called “mutational patterns” (Version 3.0.1) was used to show the mutation landscape of all samples.

**Figure 1:**
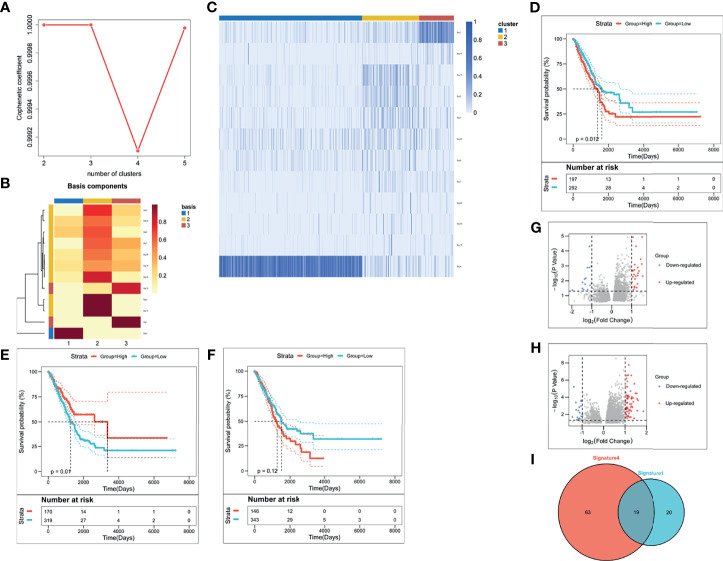
The identification of LUAD-related immune biomarkers. **(A)** The diagnostic diagram of mutation signature for setting cophenetic correlation coefficient value as three. **(B)** The evaluation of weight scores for each mutation signature in each sample by setting 6% as cut-off value. **(C)** The identification of driving mutation signatures. **(D–F)** Kaplan–Meier curves of signature 1 **(D)**, signature 4 **(E)**, and signature 13 **(F)**. **(G, H)** The selection of differentially expressed genes in signature 1 **(G)** and signature 4 **(H)**. **(I)** There were thirty-nine DEGs in signature 1 and eighty-two DEGs in signature 4.

We evaluated weight scores for each mutation signature in each sample by setting 6% as cut-off value (26) in *deconstructSigs* package (Version 1.8.0) (27). Besides, we set three as the optimal k value, according to the diagnostic diagram of mutation signature (**Figure 1A**). Consistently, the basis map (**Figure 1B**) and heatmap (**Figure 1C**) show that weight scores of signature 1, signature 4, and signature 13 were higher than others in the clustering groups 3, 1, and 2 respectively. These two figures also show that three is the optimal k value. Based on this, mutation-signature scores classified the queues into different clusters to identify driving mutation signatures by NMF consistency clustering.

Subsequently, we used *Survminer* package (Version 0.4.9) to calculate threshold values of signature weight scores. According to these threshold values, signature weight scores were divided into high group and low group. We performed survival analysis of the two groups. Limma package (Version 3.46.0) was used to calculate differential genes. Those differential genes with |log2FC| values > 1 and with false discovery rate (FDR) < 0.05 were selected for subsequent analysis. Considered as statistically significant is *p <0.05* in the survival curves. Prognostic factors that predicted poor survival were potential LUAD-related immune biomarkers. Finally, we calculated the mutation frequency of these potential LUAD-related immune biomarkers by *maftools* package (28). If the mutation frequency of those potential LUAD-related immune biomarkers is greater than 2%, these biomarkers would be finally recognized as LUAD-related immune biomarkers.

### TIMER Analysis

We applied Tumor Immune Estimation Resource (TIMER) (https://cistrome.shinyapps.io/timer/) to the investigation of the interactions between tumor immune-infiltrating cells and potential immune biomarkers for LUAD by the module for gene expression. TIMER provides an interface for dynamic analysis and visualization of the interaction (29). We conducted purity adjustment for Spearman’s correlation analysis. Defined as significant difference is *p <0.05*.

### Identification of Immune Subtypes

Based on the consensus clustering of 2,212 immune genes in LUAD patients, we used the unsupervised clustering *Pam* method by applying the *ConsensuClusterPlus* R package (Version 1.54.0) (30). To guarantee the classification stability, the method was repeated 1,000 times. Also, cluster sets varied from 2 to 10. We defined the optimal partition by identifying the consensus matrix and corresponding cumulative distribution function. Subsequently, we conducted 1,000 bootstraps in the discovery cohort and defined the optional partition by identifying the consensus matrix and corresponding cumulative distribution function. By setting the same parameters, we validated the immune subtypes in TCGA cohort. Finally, we calculated Pearson correlation and intra-group proportion in the centroids of gene module scores to qualify the immune-subtype consistency of the discovery and validation cohorts.

### Prognostic Analysis of Immune Subtypes

To calculate the prognostic values of immune subtypes, we drew Kaplan–Meier survival curves to calculate the differences of patients’ OS. We defined stage as covariates and regarded survival probability as endpoints. Next, we used ssGSEA to calculate enrichment scores of immune cells. Additionally, we performed Wilcox test to measure the differences of immune cell infiltration among immune subtypes. Single-sample gene set enrichment analysis (ssGSEA) indicated the enrichment scores of immune-filtrating cells which stand for the upregulation and downregulation of genes in one sample.

### The Description of Immune Landscape

To understand the distribution of immune subtypes in LUAD patients, we conducted dimensionality reduction analysis with the reduce dimension function of *monocle* package (Version 2.18.0) with a Gaussian distribution (31). We set two as the maximum number of components (31) and used the discriminative dimensionality reduction with trees. The final description of immune landscape was presented with cell trajectory of which cells were colored to indicate different immune subtypes.

## Results

### Mutation Patterns of LUAD and the Identification of LUAD-Related Immune Biomarkers

**Figure 2:**
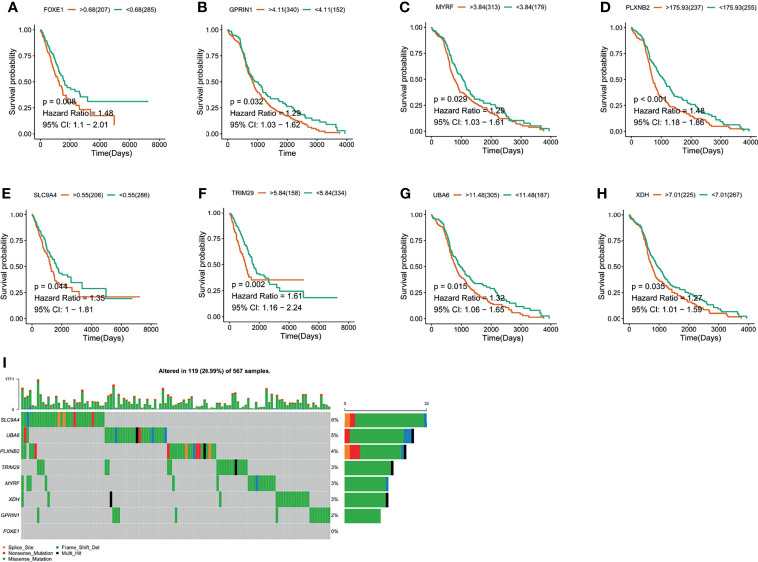
The validation of LUAD-related immune biomarkers by predicting prognosis. **(A**–**H)** Kaplan–Meier curves of LUAD patients who have mutations in FOXE1 **(A)**, GPRIN1 **(B)**, MYRF, **(C)**, PLXNB2 **(D)**, SLC9A4 **(E)**, TRIM29 **(F)**, UBA6 **(G)**, and XDH **(H)**. **(I)** Potential tumor biomarker-related gene (494 samples) with high mutation in LUAD.

Screening out LUAD-specific immune biomarkers relies on the understanding of LUAD mutation patterns (32). **Supplementary Figure 2** depicted the mutation pattern for each of the LUAD patients by identifying mutation numbers of 96 trinucleotide changes. LUAD patients are classified into four signatures: T >C, APOBEC mutation, age, and tobacco. Overall, the most common base identified in the 567 LUAD sample of TCGA was C >T. In a group with T >C signature, the common base mutations are C >G, C >T, T >C, and T >G. As for signatures of APOBEC mutation and age, C >T is the only common base change in signatures of APOBEC mutation and age. For the tobacco signature, C >A is the most frequent base change. **Figure 2B** showed the activity of four signatures among 567 LUAD. Tobacco occupies the most proportion of four signatures.

To identify driving mutation signatures, we set cophenetic correlation coefficient value as three (**Figure 1A**). By setting 6% as cut-off value (**Figure 1B**), twelve signatures were included in the module. Patients can be clustered into three subgroups based on the weight scores of these 12 signatures in the identification of driving mutation signatures by NMF consistency clustering. Signature 1, signature 4, and signature 13 are driving mutation signatures, because their weight scores were higher than others in the clustering groups 3, 1, and 2 respectively (**Figure 1C**). To further analyze the prognostic value of three signatures, we divided their signature scores into two subgroups (high group and low group), high score and low score groups which was based on the individually optimum cut-off value. High groups in signature 1 and signature 4 both indicated better survival probability than the corresponding low group (*p = 0.012* and *p = 0.01*), while there was no survival difference in signature 13 (*p = 0.12*) (**Figures 1D–F**). Finally, we chose signature 1 and signature 4 to select differentially expressed genes (DEGs). **Figure 1G** depicted upregulated and downregulated DEGs of signature 1 and **Figure 1H** depicted upregulated and downregulated DEGs of signature 4. A total of 102 DEGs were used to screen out LUAD-related immune biomarkers. There were thirty-nine DEGs in signature 1 and eighty-two DEGs in signature 4 (**Figure 1I**).

### The Validation of LUAD-Related Immune Biomarkers by Predicting Prognosis and Immune-Infiltrating Cells

**Figure 3:**
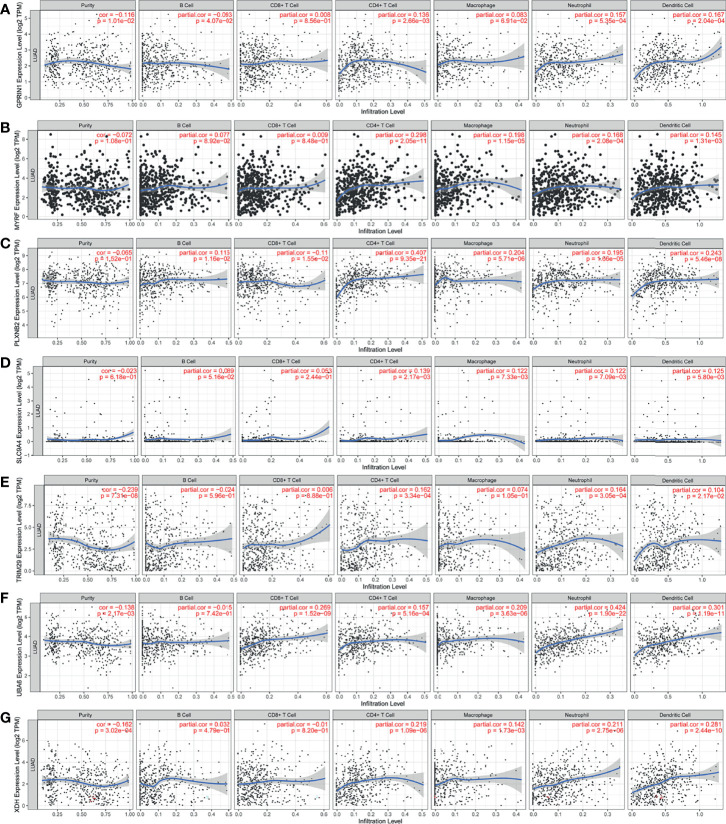
The validation of LUAD-related immune biomarkers by predicting immune-infiltrating cells. The correlation between gene expression of GPRIN1 **(A)**, MYRF **(B)**, PLXNB2 **(C)**, SLC9A4 **(D)**, TRIM29 **(E)**, UBA6 **(F)**, XDH **(G)**, and the infiltration of B cells, CD8^+^ T cells, CD4^+^ T cells, macrophages, neutrophils and DCs in LUAD.

The LUAD-related immune biomarkers used for predicting prognosis were identified to be potential immune biomarkers from the above 102 genes. In **Figures 2A–H**, patients who overexpressed and had mutations in Forkhead box protein E1 (FOXE1), G protein regulated inducer of neuriteoutgrowth 1 (GPRIN1), myelin regulatory factor (MYRF), plexin B2 (PLXNB2), solute carrier family 9 member A4 (SLC9A4), tripartite motif-containing 29 protein (TRIM29), ubiquitin-like modifier-activating enzyme 6 (UBA6), and xanthine dehydrogenase (XDH) showed poor survival probability and were correlated with poor prognosis. Collectively, these eight gene candidates were identified to be significant for LUAD progression. Furthermore, we applied maftools package to screen out biomarker-related genes whose mutation frequency is over 2%. They were SLC9A4, UBA6, PLXNB2, TRIM29, MYRF, XDH, GPRIN1, and FOXE1 (**Figure 2I**). However, FOXE1 mutation was not detected. These genes are candidates for target genes. Furthermore, tumor purity determines the detection of somatic mutation and the pattern of gene expression. Based on this confounding factor, we checked *purity adjustment* option (29, 33). After purity adjustment, up-regulation of GPRIN1, MYRF, PLXNB2, SLC9A4, TRIM29, UBA6, and XDH positively correlated with the infiltration of CD4^+^ T cells, neutrophils, and dendritic cells (DCs). PLXNB2 and UBA6 showed positive correlation with CD8^+^ T cells. Besides, GPRIN1 and PLXNB2 were positively correlated with B cells. As for macrophages, except patients with high GPRIN1 and TRIM29, the rest five kinds of patients had high levels (**Figures 3A–G**). These results suggested that these LUAD-associated immune biomarkers were presented by antigen-presenting cells (macrophages and DCs) to activate T cells and B cells for initiating the immune response. Therefore, GPRIN1, MYRF, PLXNB2, SLC9A4, TRIM29, UBA6, and XDH are potential LUAD-related immune biomarker candidates to activate the immune response.

### The Identification of LUAD-Related Immune Subtypes

**Figure 4:**
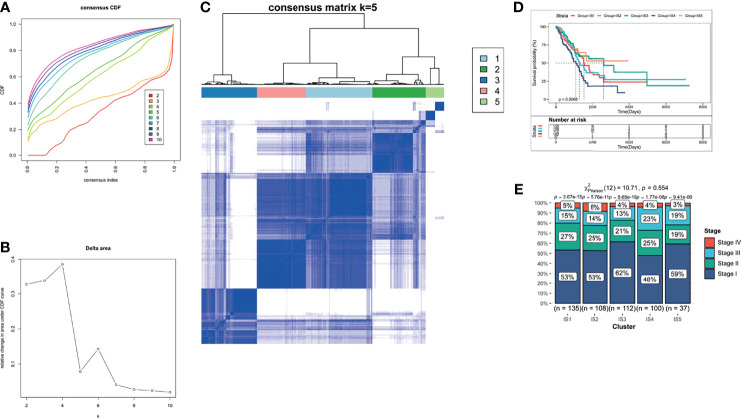
The identification of LUAD-related ISs. **(A)** Cumulative distribution function curves. **(B)** Delta area of immune-related genes. **(C)** Consensus matrix heatmap for sample clustering. **(D)** Kaplan–Meier curves of LUAD patients in different ISs. **(E)** Distribution of LUAD IS1-IS5 in four stages (Stages I–IV).

The therapeutic effect of mRNA vaccination relies on innate immunity and adaptive immunity. However, some of the cancer patients’ immune system is impaired. Therefore, the immune status of cancer patients is a crucial factor to assess the therapeutic efficacy. We analyzed the expression levels of 2,212 immune genes to build a consensus matrix. The classifier model is the most stable when we set k as 5 (34). Hence, we clustered five LUAD-related immune subtypes (IS) (**Figures 4A, B** **)** and named every subtype as 1–5 (**Figure 4C**). Compared with patients in IS1, IS2, IS4, and IS5, patients in IS3 showed prolonged survival probability (**Figure 4D**). Furthermore, five LUAD-related immune subtypes clustered LUAD stages. All immune subtypes contain a large proportion of stage 1 patients which occupied 48–62% in every immune subtype (**Figure 4E**). Therefore, LUAD-related immune subtypes show potential superiority in predicting prognosis, compared with prevalent tumor staging.

### The Correlation of LUAD-Related Immune Subtypes and Mutation

**Figure 5:**
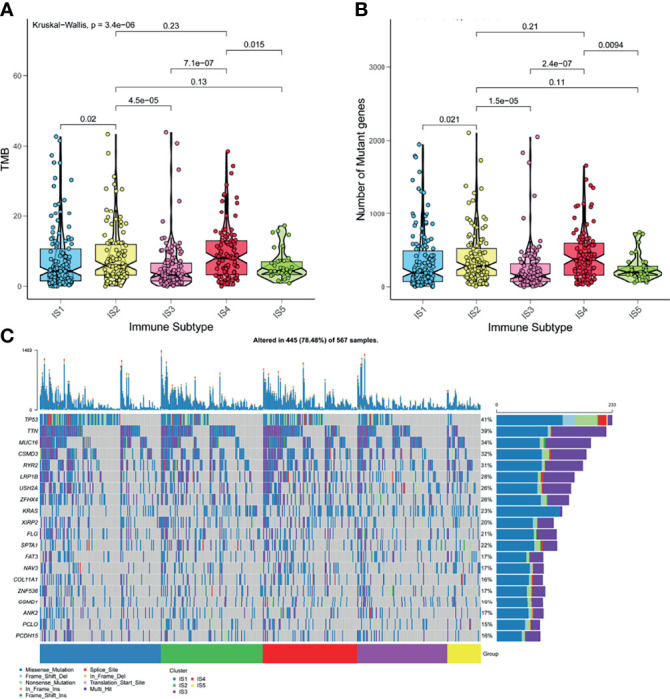
The correlation of LUAD-related ISs and mutation. **(A)** TMB in five ISISs. **(B)** The number of somatic mutations in five ISISs. **(C)** The distribution of nineteen highly mutated genes in five ISISs of LUAD.

High tumor mutation burden (TMB) and increased number of somatic mutations are associated with anti-cancer effects. Theoretically, higher TMB promotes the production of neoantigens, facilitating immune recognition and cancerous killing (35). Based on the theory, we analyzed the TMB and the number of somatic mutations in patients from the mutation dataset of TCGA. **Figure 5A** indicated that patients with IS2 and IS4 have higher TMB, compared with other immune subtypes. Consistent results were observed in the number of mutant genes (**Figure 5B**). IS2 and IS4 comprised the largest number of somatic mutations. Moreover, patients in IS2 and IS4 harbored more proportion of mutation status than other patients (**Figure 5C**). Together, these findings suggested that LUAD-related immune subtypes could predict TMB, the number of somatic mutations.

### The Correlation of LUAD-Related Immune Subtypes and Immune Modulators

**Figure 6:**
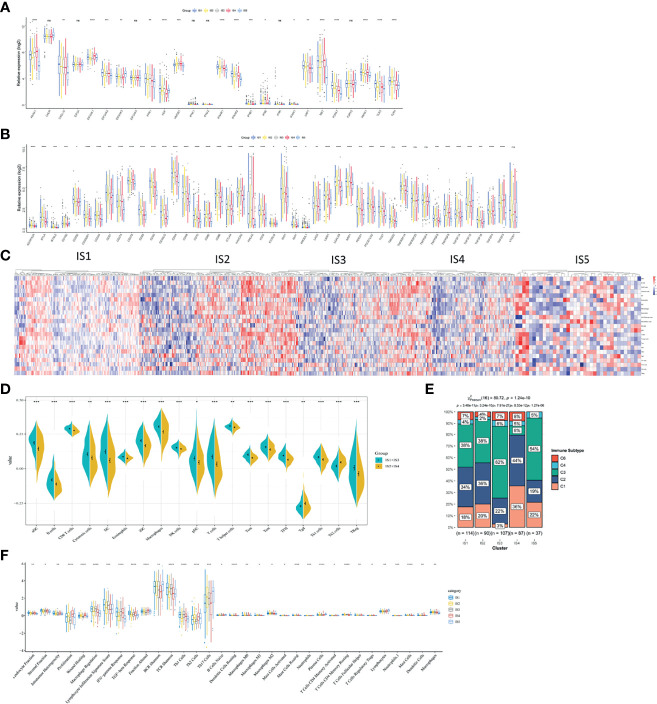
The correlation between LUAD-related ISs and immune modulators and the feature of immune cells in different ISs. **(A)** Differentially expressed ICP genes in different LUAD ISs. **(B)** Differentially expressed ICD genes in different LUAD ISs. Kruskal–Wallis test with ^*^ *p <* 0.05, ^**^ *p <* 0.01, ^***^ *p <* 0.001, ^****^ *p <* 0.001. *ns* presents no difference. **(C)** Differential enrichment scores of immune cell signatures among five ISs in TCGA cohorts. **(D)** Differential enrichment scores of ten prognosis-related immune cell signatures. **(E)** The distribution of five pan-cancer ISs in five LUAD-related ISs. **(F)** Differential enrichment scores of twenty-four immune signatures.

The polymorphism of genes encoding immune modulators is associated with therapeutic effects (36). As an indicator of immune response, some immune modulators attached great importance to cancer therapy, such as immune checkpoint (ICP) (37) and immunogenic cell death (ICD) (38). Therefore, we analyzed the expression levels of ICP-related and ICD-related modulatory genes in TCGA database. Forty-five ICP-related genes was detected and forty-one genes were differentially expressed among five immune subtypes. Kruskal–Wallis test was used to detect differences of gene expression among five independent ISs. For the genes with *p <*0.05, the gene expression was significantly different among the five ISs. ADORA2A, BTLA, BTNL2, CD160, CD200, CD200R1, CD244, CD27, CD28, CD40, CD40LG, CD44, CD48, CTLA4, HHLA2, ICOS, ICOSLG, IDO1, IDO2, LAG3, LAIR1, LGAL59, PDCD1, TIGIT, TNFRSF12, TNFRSF8, TNFRSF9, TNFSF14, and TNFSF18 were all overexpressed in LUAD with IS3 (**Figure 6A**). As for ICD-related modulatory genes, twenty-six genes were detected and twenty-two genes were differentially expressed among five ISs by Kruskal–Wallis test. ANXA1, CXCL10, EIF2AK1, EIF2AK2, EIF2AK3, FPR1, HGF, HMGB1, IFNAR1, IFNAR2, LRP1, MET, P2RY7, PANX1, TLR3, and TLR4 were all upregulated in LUAD with IS1 (**Figure 6B**). Therefore, LUAD-related immune subtypes represent the expression levels of immune modulators, which are representative biomarkers of therapeutic effects of mRNA vaccines.

### The Feature of Immune Cells in Different Immune Subtypes

Vaccine response is predicted by immune status. Therefore, we analyzed different kinds of immune cells in five immune subtypes by calculating the enrichment scores of 23 signature genes (39) with ssGSEA. Five clusters of immune cells can be grouped into two parts, according to similar immune-cell scores (**Figure 6C**). One is composed of IS1 and IS3 and the other contains IS2 and IS4. The integral distribution of IS1 and IS3 was opposite to that of IS2 and IS4. Compared with patients with IS2 and IS4, aDC, B cells, CD8^+^ T cells, cytotoxic cells, DCs, eosinophils, iDCs, macrophages, NK cells, pDCs, T cells, T helper cells, Tcm, Tem, TFH, Th1 cells, and Treg cells were all significant abundant in patients with IS1 and IS3. In patients with IS2 and IS4, only Th2 cells were enriched (**Figure 6D**). Hence, IS1 and IS3 stand for *hot* immunity, while IS2 and IS4 stand for *cold* immunity. *Hot* immunity represents an increased immune cell infiltration, which is interpreted as an inflamed microenvironment (40). Inflammation is positively correlated with tumor progression and immunosuppressive microenvironment (41). Similarly, *cold* immunity means decreased immune cell infiltration. Our findings indicated that patients with IS2 and IS4 were suitable for mRNA vaccination. mRNA vaccines have the potential to generate immune-infiltrating cells in patients with IS2 and IS4.

Thorsson et al. have identified five immune subtypes of LUAD (C1–C4 and C6) (42). To validate the reliability of our five immune subtypes, we comprehensively analyzed the relationship of C1–C6 and IS1–IS5. IS1, IS2, IS3, and IS5 both encompassed C2 and C3 for at least 19 and 38% respectively. IS3 contains all the five immune subtypes and overlapped with C3 for 62% (**Figure 6E**). Thorsson et al. reported that C3 had the best prognosis. The prognostic conditions of C1 and C2 is inferior to that of C3. C4 and C6 had the worst clinical outcomes (42). Our previous results proved that patients in IS3 showed prolonged OS (**Figure 4D**). Due to 62% of C3 in IS3 and 22% of C2 in IS3, patients with IS3 tend to have good clinical outcomes. In sum, our findings validated the reliability of our five immune subtypes, which was explained by Thorsson’s results.

Finally, we characterized the feature of immune cells in different immune subtypes by analyzing 56 previously detected molecular signatures. Significantly identified were 33 out of 56 immune-related signatures. In **Figure 6F**, IS1 scored high in leukocyte fraction, stromal fraction, intratumor heterogeneity, macrophage regulation, lymphocyte infiltration signature score, IFN-γ response, TGF-β response, fraction altered, BCR Shannon, TCR Shannon, Th1 cells, dendritic cells resting, macrophages M0, macrophages M1, mast cells resting, T cells CD4 memory resting, T cells regulatory Tregs, mast cell, dendritic cells, and macrophages. However, IS1 scored low in lymphocytes. Hence, IS1 was an active immune-related phenotype with an enrichment of positively regulated immune cells. When it comes to IS3, IS3 scored high in macrophage regulation, lymphocyte infiltration signature score, IFN-γ response, TCR Shannon, Th1 cells, and macrophages. As for IS4, low scores of macrophage regulation, lymphocyte infiltration signature score, BCR Shannon, TCR Shannon, Th17 cells, B cells naïve, macrophage M2, mast cells resting, plasma cells, T cells CD4 memory resting, mast cells, and macrophages stands for *cold* immunity. Hence, these cells in IS4 formed an immunosuppressive tumor microenvironment and indicating *cold* immunity. Also, IS2 showed moderate infiltration with increased stromal fraction and active TGF-β response, which is an immunosuppressive phenotype.

Collectively, different immune subtypes encompassed the characteristics of molecules and immune cells and showed corresponding immune status. Therefore, patients with IS2 and IS4 (*cold* immunity) are ideal vaccination recipients.

### The Immune Landscape of LUAD

**Figure 7:**
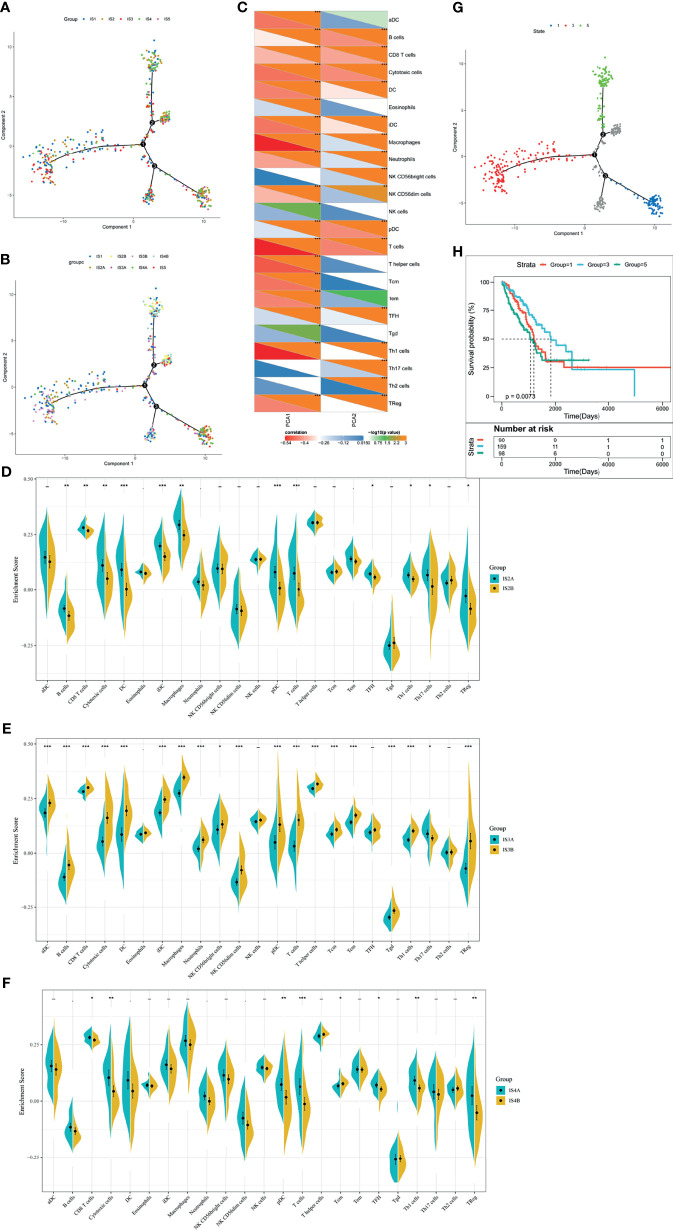
The immune landscape of LUAD. **(A)** Dimensionality reduction analysis of LUAD patients. Each point stands for a patient with a certain IS. The horizontal axis represents PCA1 and the vertical axis represents PCA2. **(B)** Immune landscape of the subgroups of LUAD-related ISs. **(C)** The correlation heatmap of PCA1 and PCA2 with 23 immune cell signatures. **(D**–**F)** Differential enrichment scores of twenty-three immune cell signatures in the IS3 **(D)**, IS4 **(E)**, and IS5 **(F)**. *p < 0.05, **p < 0.01, ***p < 0.001. **(G)** Immune landscape of samples from three extreme bivariate distributional positions (groups 1, 4, and 5). **(H)** Kaplan–Meier curves of LUAD patients in three extreme bivariate distributional positions.

To realize the individualized therapy, immune landscape is essential to describe the immune components of LUAD patients. We described the immune landscape of LUAD by analyzing patients’ expression levels of immune genes (**Figure 7A**). The horizontal ordinate was negatively correlated with Treg and positively correlated with NK CD56^bright^ cells. The longitudinal coordinate was negatively associated with B cells, CD8 T cells and cytotoxic cells (**Figure 7C**). S2, S3, and S4 separated apart in the bivariate distribution. Points of S1 and S5 scattered throughout the bivariate area, suggesting that there is heterogenicity within the immune subtypes (**Figure 7B**). Moreover, we divided S2, S3, and S4 into two subgroups (A and B), respectively. Some immune cells of the two subgroups in a certain immune subtype were significantly different from each other. **Figure 7D** depicted that IS2B had less amount of B cells, CD8^+^ T cells, cytotoxic cells, DC, iDC, macrophage, pDC, T cells, TFH, Th1 cells, Th7 cells and Treg, compared with IS2A. In **Figure 7E**, IS3A scored lower in aDC, B cells, CD8^+^ T cells, cytotoxic cells, DC, iDC, macrophage, neutrophils, NK CD56^bright^ cells, NK CD56^dim^ cells, pDC, T cells, T helper cells, Tcm, Tem, Tgd, Th1 cells, Th17 cells, and Treg. Similar with IS2B, IS4B have lower scores of CD8^+^ T cells, cytotoxic cells, pDC, T cells, Tcm, TFH, Th1 cells, and Treg (**Figure 7F**). Hence, mRNA vaccine is effective in patients with IS2B, IS3A, and IS5B. Furthermore, we classified each point according to their extreme bivariate distributional position and selected groups 1, 3, and 5 for survival analysis. Group 3 showed the best survival probability (**Figures 7G, H** **)**.

In this section, we used immune subtypes to describe the landscape of LUAD. LUAD immune landscape revealed immune cells and prognostic conditions, which provides important information to select patients for individualized therapy.

### The Analysis of LUAD Immune-Gene Co-Expression Networks and LUAD-Related Hub Genes

**Figure 8:**
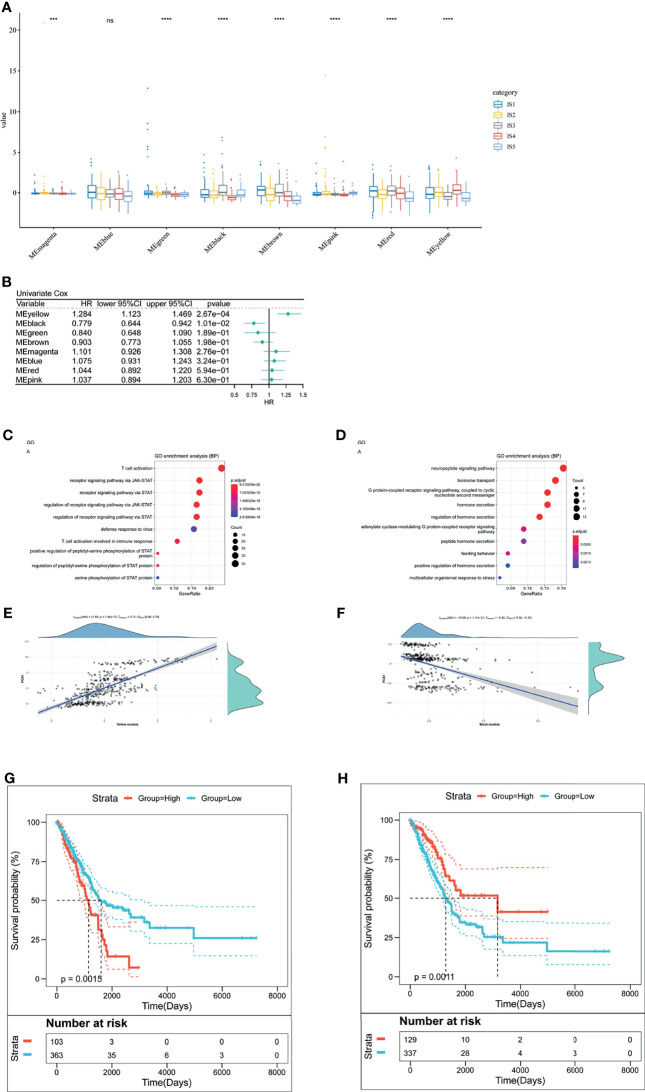
The analysis of LUAD-related hub genes. **(A)** Differential distribution of five LUAD-related ISs in each module. **(B)** Forest maps of survival analysis of eight LUAD modules. ***p < 0.001, ****p < 0.001. *ns* presents no difference. **(B, C)** Enrichment map of biological processes in the yellow module **(C)**. The dot size stands for the number of events. The color intensity stands for the enrichment level. The correlation between the yellow module and PCA1 component in immune landscape **(D)**. **(E, F)** Enrichment map of biological processes in the black module **(E)**. The dot size stands for the number of enrichment genes. The color intensity stands for the enrichment level. The correlation between the black module and PCA1 component in immune landscape **(F)**. **(G)** Kaplan–Meier curves of the yellow module with low group scores and high group scores. **(H)** Kaplan–Meier curves of the black module with low group scores and high group scores.

We clustered samples by WGCNA (**Supplementary Figure 4A**) and created LUAD immune-gene co-expression networks by setting four as a threshold for scale-free network (**Supplementary Figure 4B**). Followed by the convert of an adjacency matrix from the representation matrix, the adjacency was further converted to a topological matrix. Hierarchy clustering was carried out using thirty genes for each network. Then, we extracted cluster eigengenes and integrated similar clusters into one cluster whose height was 0.25, deep split was 4, and minimum module size is 30. Next, we identified nine LUAD immune-gene co-expression modules of 2,212 immune genes (**Supplementary Figure 4C**). **Supplementary Figure 4D** showed eight clustered co-expression modules with different colors. Finally, we analyzed the differential distribution of five immune subtypes among eight modules and five immune subtypes in eight modules were differentially distributed (**Figure 8A**). IS3 had high eigengenes in MEblack and MEred.

Furthermore, to identify LUAD-related hub genes, we predicted that yellow and black modules were associated with LUAD prognosis (**Figure 8B**). The yellow module related to T cell activation and positively correlated with the horizontal component (PCA1) of immune landscape positively (**Figures 8C, E** **)**. Likewise, the black module had correlation with G protein-coupled receptor signaling pathway and negatively correlated with PCA1 (**Figures 8D, F** **)**. Next, we analyzed the prognosis-related genes in the yellow module and the black one, respectively. Survival curves of the yellow module indicated that the group with low scores was associated with increased survival probability, due to the negative correlation of PCA1 and Treg (**Figure 8G**). In contrast, Survival curves of the black module suggested better survival probability in the group with high scores (**Figure 8H**). It commonly accepted that the inhibition of immune-suppressive cells (Treg) results in the therapeutic efficacy of vaccines. Given the potential of mRNA vaccines in activating immune responses, mRNA vaccines are suitable for low-group-score patients that were clustered into the yellow module and the black one. LUAD-related hub genes were IFNK, MATR3, ATP8B4, ANGPTL7, IFIT1B, PVRIG (yellow module), GRP, and CHGA (black module).

## Discussion

Our study is the first identification of immune biomarkers and immune subtypes of LUAD. We depicted the mutation pattern of LUAD, identified LUAD-related immune biomarkers and immune subtypes and analyzed the correlation of LUAD-related immune subtypes and mutation. Besides, we further analyzed the correlation of LUAD-related immune subtypes and immune modulators, discovered the feature of immune cells in different immune subtypes, depicted the immune landscape of LUAD and finally identified LUAD immune-gene co-expression networks and LUAD-related hub genes. GPRIN1, MYRF, PLXNB2, SLC9A4, TRIM29, UBA6, and XDH are all mRNA vaccine candidates. They positively correlated with poor survival probability and the filtration of CD4^+^ T cells, CD8^+^ T cells, B cells, macrophages, and DCs. Therefore, these LUAD-associated immune biomarkers were presented by antigen-presenting cells (macrophages and DCs) to activate T cells for initiating the immune response. Accumulated evidence has confirmed their potential in inhibiting tumor growth. Firstly, GPRIN1 is overexpressed in lung cancer cells. Detarya et al. inhibited GPRIN1 expression in A549 cell lines, observing attenuated biological process and mesenchymal transition (EMT)-related phenotypes (43). Secondly, MYPF (MYRF) is an upregulated ER membrane-associated transcription factor in pancreatic ductal adenocarcinomas. Well-differentiated secretory cancer cells contain a large quantity of MYRF, while MYRF is absent in poorly differentiated quasi-mesenchymal cells. Mechanically, MYRF participates in the expression of genes encoding highly glycosylated, cysteine-rich secretory proteins, which is regulated by HNF1B. The role of MYRF is to prevent ER stress. To validate the ER-stress prevention of MYRF, Milan et al. discovered ER stress, inhibited proliferation and inability to form spheroids *in vitro* in MYRF-deficient pancreatic ductal adenocarcinoma cells. *In vivo*, the cells formed a less proliferating tumor (44). Then, PLXNB2 is highly expressed on B cells from germinal center (GC). It recruits GC follicular T helper cells and optimize antibody responses. By removing PLXNB2 from the DC, interactions of T cells and B cells, and plasma cell production/affinity maturation were impaired (45). Lin et al. found that circPLXNB2 (a circular RNA) and PLXNB2 mRNAs were high in patients with acute myeloid leukemia (AML). Those patients had poor OS and disease-free survival (PFS), which is consistent with our study. They also indicated that PLXNB2 promoted proliferation and migration and that suppressed apoptosis both *in vivo* and *in vitro* (46). Next, SLC9A4 is also known as sodium–hydrogen exchanger (NHE) 4 protein. SLC9A4 is reported to exist in T84 human colon cancer cell, which occupied 43% of pH recovery after an acid intervention (47). Besides, TRIM29 promoted EMT-mediated invasion and metastasis of colorectal cancer by activating Wnt/β-catenin signaling pathway (48). Similar research indicated that TRIM29 overexpression facilitated the proliferation of bladder cancer cells by the activation of NF-κB (49). Zhou et al. analyzed the relationship between TRIM29 and β-catenin in patients with non-small-cell lung cancer, discovering that TRIM29 was overexpressed in adenocarcinoma of non-small-cell lung cancer (50). Furthermore, UBA6 is ubiquitin-activating enzyme which cooperated with hybrid ubiquitin-conjugating enzyme/ubiquitin ligase (BIRC6) to limit LC3B for autophagy (51). A relevant study investigated UBA6-specific E2 conjugating enzyme 1 (USE1). The results showed that mutations in USE1 resulted in lung tumorigenesis by prolonging the half-life of the protein (52). We believe that USE1-associated UBA6 might be a participator in the prognosis of LUAD patients. Finally, the balance of oxidase and xanthine dehydrogenase (XDH) determines the tumor growth. Xu et al. discovered that xanthine oxidase-mediated oxidative stress promotes the apoptosis of tumor cells (53). In other words, XDH might favor tumor growth. XDH were identified as a target of oncogenic steroid receptor coactivator-3 (SRC-3) (54). Targeting XDH would be a suppressive strategy to treat LUAD.

Certain population of LUAD responds effectively to mRNA vaccines. We classified the immune status of LUAD patients into five immune subtypes, hoping to select suitable population for vaccination. Different immune subtypes have different cellular/molecular characteristics and clinical outcomes. For more detail, patients in IS3 both showed prolonged clinical outcomes in TCGA database. Such a result implies that LUAD-related immune subtypes show potential superiority in predicting prognosis, compared with prevalent tumor staging. Another value of our study is that immune subtypes are a representative of therapeutic reactivity and efficacy. Patients with IS2 and IS4 have the highest TMB and the largest number of somatic mutations, which means that they have robustly responsive reaction to mRNA vaccines. ICP-related genes were overexpressed in LUAD with IS3, indicating an immunosuppressive tumor microenvironment. For those patients, robust immune responses are difficult to be induced by mRNA vaccines. Differently, the upregulated ICD-related modulatory genes are a positive indicator of therapeutic effects of mRNA vaccines in LUAD with IS1. Furthermore, the third value of our study is the development of individualized therapy by depicting the immune landscape of LUAD. Individual difference originates from the complicated immune landscape. By analyzing immune cells and prognostic conditions of each patient, we would select patients for individualized therapy. Finally, LUAD-related hub genes (IFNK, MATR3, ATP8B4, ANGPTL7, IFIT1B, and PVRIG) in the yellow module and those (GRP and CHGA) in the black module are positively and negatively correlated with PCA1 of immune landscape, respectively. Patients harboring these elevated genes are responsive to mRNA vaccines. Low-group-score patients in the yellow module and the black one are promising LUAD-mRNA-vaccine candidates.

Effective vaccination response is reflected by immune status. Therefore, we identified the exact immune cells in five immune subtypes. Patients with IS1 and IS3 had more aDC, B cells, CD8+ T cells, cytotoxic cells, DCs, eosinophils, iDCs, macrophages, NK cells, pDCs, T cells, T helper cells, Tcm, Tem, TFH, Th1 cells, and Treg cells, compared with patients with IS2 and IS4. Patients with IS2 and IS4 included more Th2 than those with IS1 and IS3. Hence, IS1 and IS3 stand for hot immunity, while IS2 and IS4 stand for cold immunity. These immune signatures correspond to their molecular features. For example, IS2 showed moderate infiltration with increased stromal fraction and active TGF-β response. IS4 scored low in macrophage regulation, lymphocyte infiltration signature score, BCR Shannon, TCR Shannon, Th17 cells, B cells naïve, macrophage M2, mast cells resting, plasma cells, T cells CD4 memory resting, mast cells, and macrophages. Cells in IS2 and IS4 formed an immunosuppressive tumor microenvironment. To increase the therapeutic reactiveness for innate immunity and adaptive immunity, mRNA vaccines is an ideal candidate to produce immunity-activating immune biomarkers. Moreover, the combined therapy of ICP blockage/ICD-related modulators and mRNA vaccines would strengthen the therapeutic effects by increasing the immune-infiltrating cells. These patients might work for the combined therapy of ICP blockage/ICD-related modulators and mRNA vaccines.

Thorsson et al. classified LUAD into C1–C4 and C6. C3 had the best prognosis, while C4 and C6 had the worst clinical outcomes. In our study, we classified LUAD into IS1–IS5 subtypes. IS1 overlapped with C2 and C3, IS2 with C2 and C3, IS3 with C2 and C3, IS4 with C2 and C3 and IS5 with C3. These two classification methods act in accordance with each other, because we have validated good clinical outcomes in patients with IS3. Patients with IS1, IS2, and IS4 showed similar clinical outcomes. Their OS were inferior to the OS of patients with IS3. Therefore, our LUAD-related immune subtypes show potential superiority in predicting prognosis, compared with prevalent tumor staging.

Although we classified the immune status of the LUAD patients into immune subtypes for the determination of patients’ mRNA vaccine sensitivity, the limitation of our study should be noticed. mRNA sequence is a determinant fact to maintain mRNA stability, eliminate mRNA immunogenicity and exert effective immune responses (10). However, our study did not provide the sequence of potential LUAD-related immune biomarkers. The provided LUAD-related biomarker genes might contain multiple subunit biomarkers which are all possible to exert robust immunity. Future studies would focus on the mRNA sequence design of those LUAD-related immune biomarkers.

## Conclusions

GPRIN1, MYRF, PLXNB2, SLC9A4, TRIM29, UBA6, and XDH are potential LUAD-related immune biomarker candidates to activate the immune response. Patients with IS2 and IS4 might potentially be immunization-sensitive patients for vaccination. Our study validated mRNA-vaccine immune biomarker candidates for LUAD and selected appropriate patient cohorts corresponding to immune subtypes and predicting prognosis.

## Data Availability Statement

The raw data supporting the conclusions of this article will be made available by the authors, without undue reservation.

## Author Contributions

HS offered main direction and significant guidance of this manuscript. YW and HT drafted the manuscript and illustrated the figures for the manuscript. They contributed equally to the work. TY revised and check the manuscript. XC and FJ made the figure. All authors contributed to the article and approved the submitted version.

## Funding

This work was financially supported by the National Natural Science Foundation of China (Grant No. 82003195), and the China Postdoctoral Science Foundation (Grant No. 2020M680150).

## Conflict of Interest

The authors declare that the research was conducted in the absence of any commercial or financial relationships that could be construed as a potential conflict of interest.

## Publisher’s Note

All claims expressed in this article are solely those of the authors and do not necessarily represent those of their affiliated organizations, or those of the publisher, the editors and the reviewers. Any product that may be evaluated in this article, or claim that may be made by its manufacturer, is not guaranteed or endorsed by the publisher.

## Supplementary Material

Supplementary Figure 1 Flow diagram of the data processing, analysis, and validation. We downloaded clinical information and RNA-seq data of 494 LUAD patients from Cancer Genome Atlas (TCGA), followed by the identification of potential immune biomarkers, the analysis of five LUAD-related ISs and immune hub genes of PAAD. Created with BioRender.com

Supplementary Figure 2 The mutation pattern of LUAD patients. **(A)** The identification of mutation numbers of 96 trinucleotide changes. We used NMF to analyze the mutation signatures of LUAD by clustering patients’ mutation information. These mutation signatures are T>C, APOBEC mutation, age and tobacco. **(B)** The number of four mutation signatures and their proportions are analyzed according to LUAD patients’ information from TCGA cohort.

Supplementary Figure 3 The diagnostic diagram of mutation pattern for setting cophenetic correlation coefficient value as four.

Supplementary Figure 4 The analysis of LUAD immune-gene co-expression networks. **(A)** A sample clustering. **(B)** LUAD immune-gene co-expression networks by setting four as a threshold for scale-free network. **(C)** Dendrogram of all differentially expressed genes. **(D)** Gene numbers in each module.

## Abbreviations

LUAD, Lung Adenocarcinoma; PD-1, Programmed cell death-1; PD-L1, programmed cell death-1 ligand; CTLA-4, cytotoxic T-lymphocyte-associated antigen 4; COVID-19, Corona Virus Disease 2019; SOCS, suppressor of cytokine signaling; TCGA, Cancer Genome Atlas; GDC, Genomic Data Commons; FPKM, fregments per kilobase per million; TPM, transcripts per million; NMF, non-negative Matrix Factorization; FDR, false discovery rate; TIMER, tumor immune estimation resource; ssGSEA, single-sample gene set enrichment analysis; FOXE1, Forkhead box protein E1; GPRIN1, G protein regulated inducer of neurite outgrowth 1; MYRF, myelin regulatory factor; PLXNB2, plexin B2; SLC9A4, solute carrier family 9 member A4; TRIM29, tripartite motif-containing 29 protein; UBA6, ubiquitin-like modifier-activating enzyme 6; XDH, xanthine dehydrogenase; DC, dendritic cells; IS, immune subtypes; TMB, tumor mutation burden; ICP, immune checkpoint; ICD, immunogenic cell death; EMT, epithelial to mesenchymal transition; ER, endoplasmic reticulum; GC, germinal center; AML, acute myeloid leukemia; PFS, acute myeloid leukemia; USE1, UBA6-specific E2 conjugating enzyme 1; XDH, xanthine dehydrogenase; SRC-3, steroid receptor coactivator-3; DEG, differentially expressed genes.
