# Supplementary figures and images for: Potential Immune Biomarker Candidates and Immune Subtypes of Lung Adenocarcinoma for Developing mRNA Vaccines
Source: Front Immunol. 2021 Nov 30;12:755401. doi: 10.3389/fimmu.2021.755401 (PMC8670181; doi:10.3389/fimmu.2021.755401)

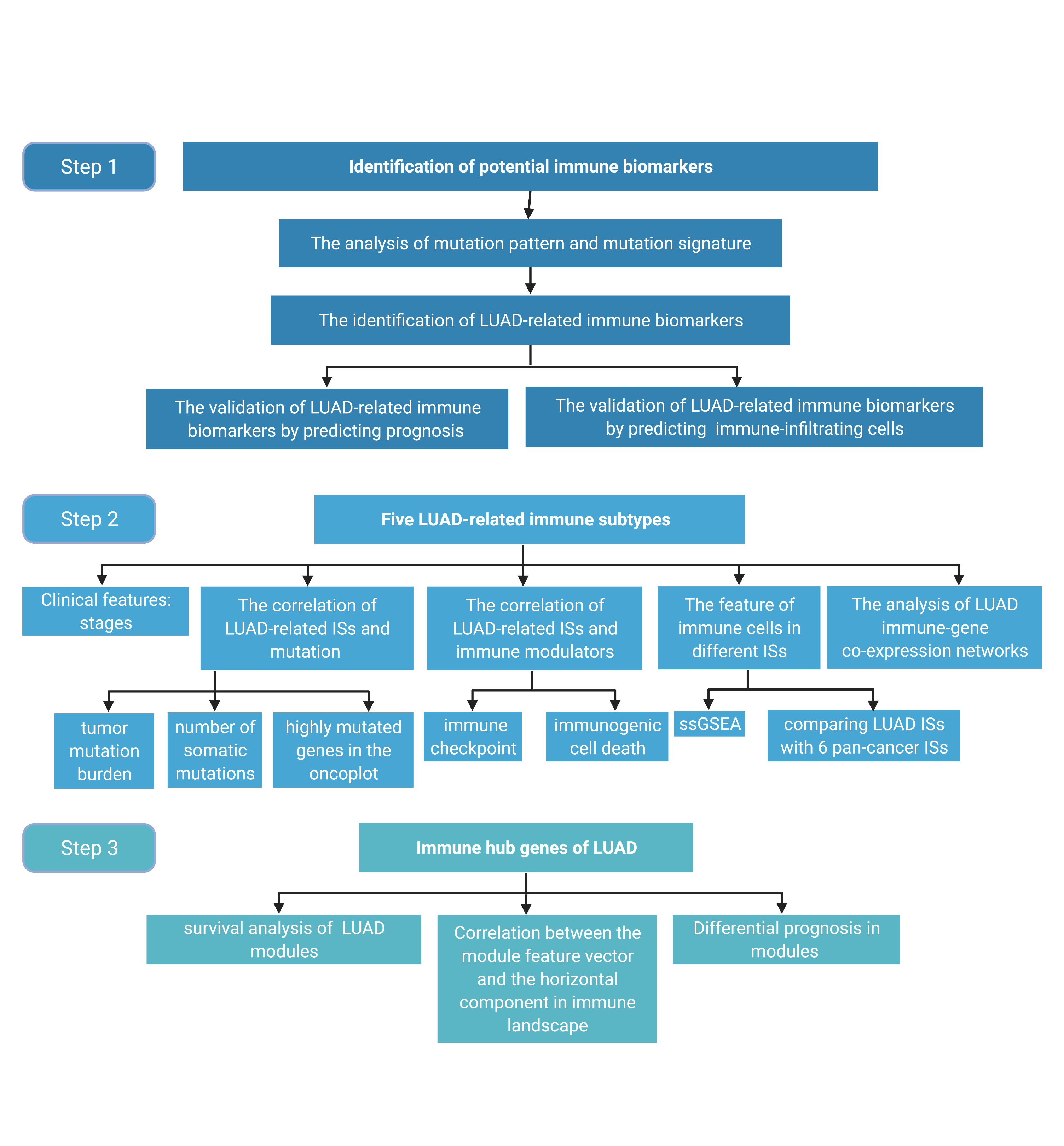

Supplement: Supplementary Figure 1 — Flow diagram of the data processing, analysis, and validation. We downloaded clinical information and RNA-seq data of 494 LUAD patients from Cancer Genome Atlas (TCGA), followed by the identification of potential immune biomarkers, the analysis of five LUAD-related ISs and immune hub genes of PAAD. Created with BioRender.com [file Image_1.jpeg]

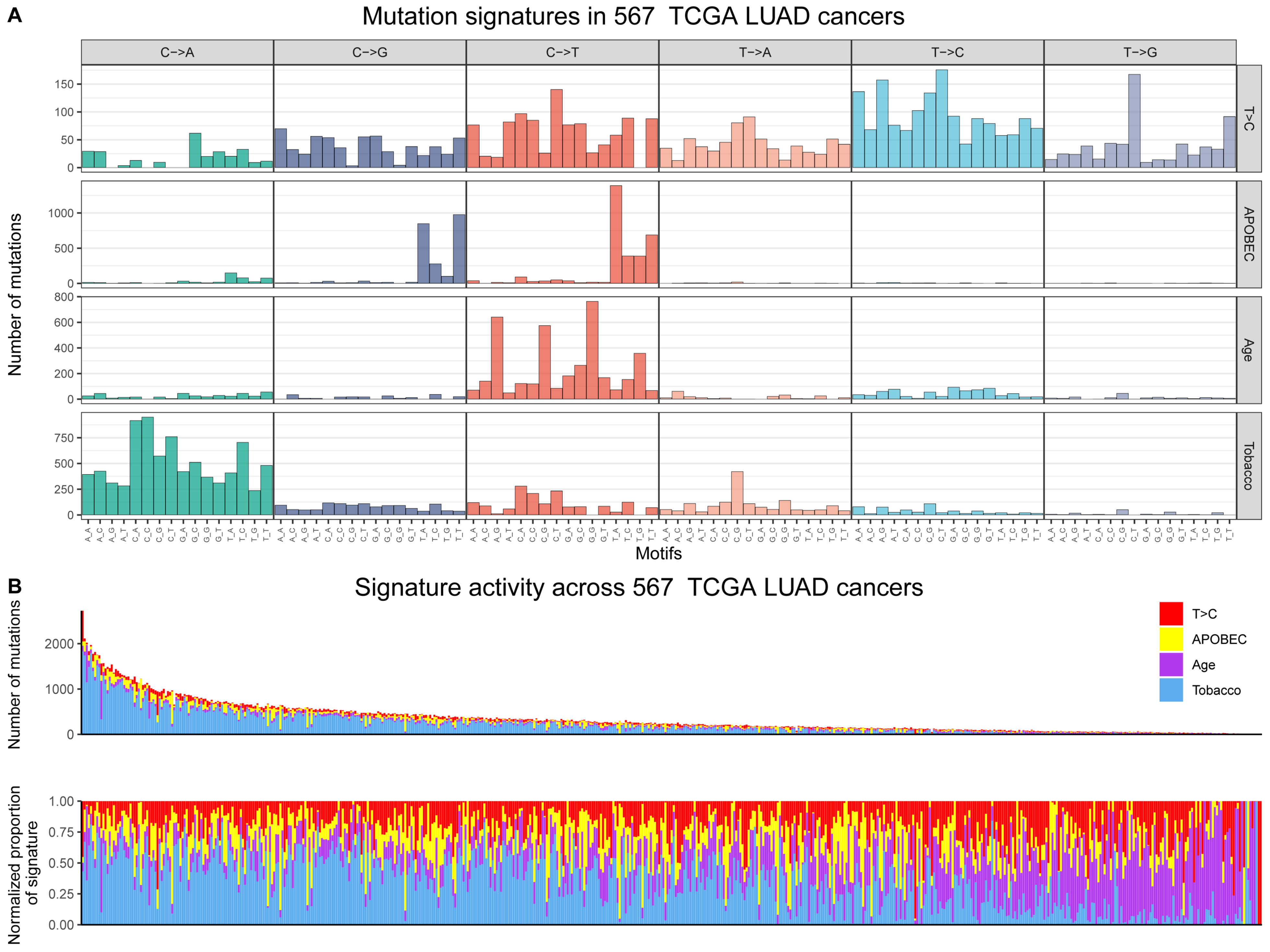

Supplement: Supplementary Figure 2 — The mutation pattern of LUAD patients. (A) The identification of mutation numbers of 96 trinucleotide changes. We used NMF to analyze the mutation signatures of LUAD by clustering patients’ mutation information. These mutation signatures are T>C, APOBEC mutation, age and tobacco. (B) The number of four mutation signatures and their proportions are analyzed according to LUAD patients’ information from TCGA cohort. [file Image_2.tif]

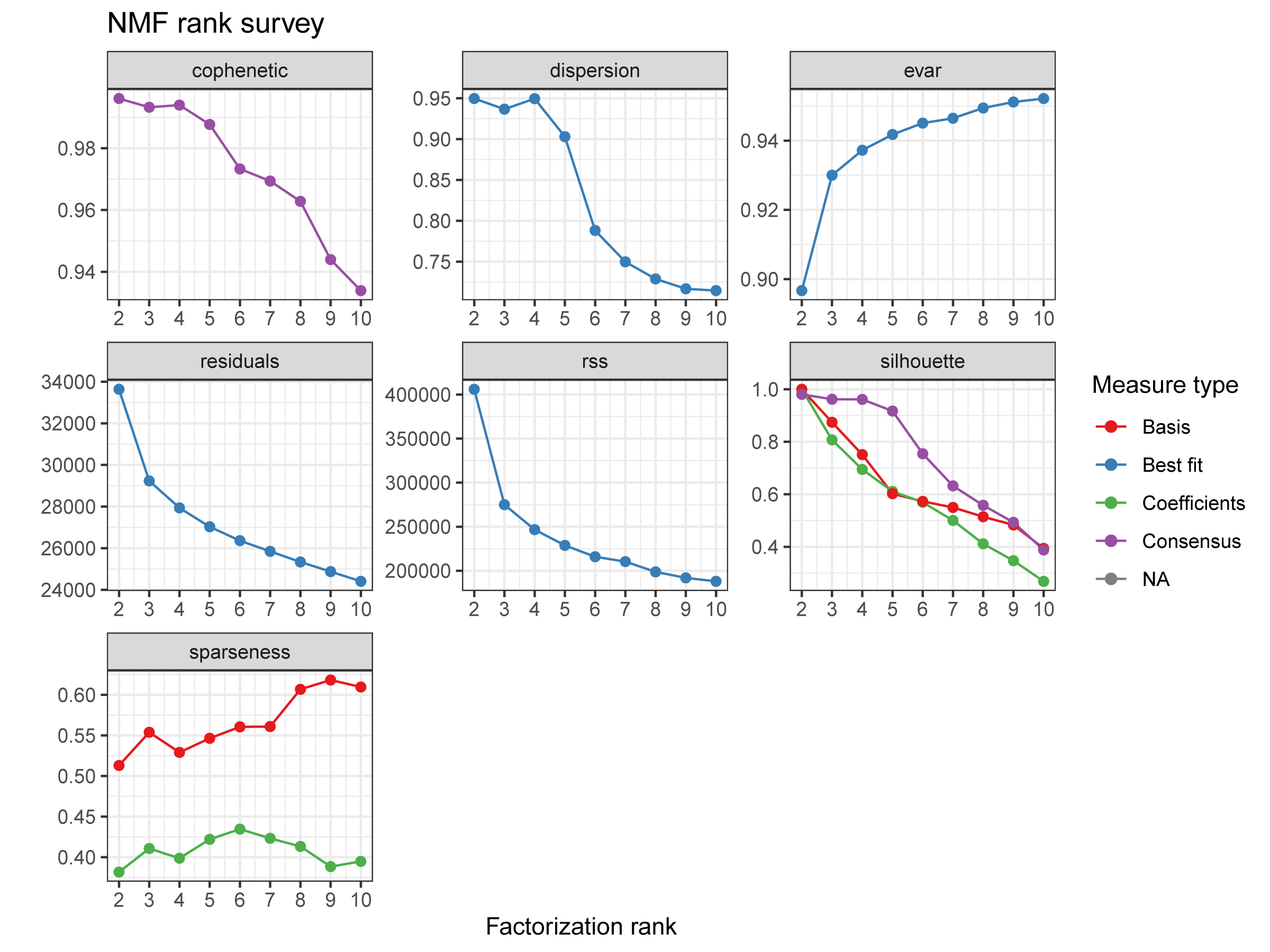

Supplement: Supplementary Figure 3 — The diagnostic diagram of mutation pattern for setting cophenetic correlation coefficient value as four. [file Image_3.tif]

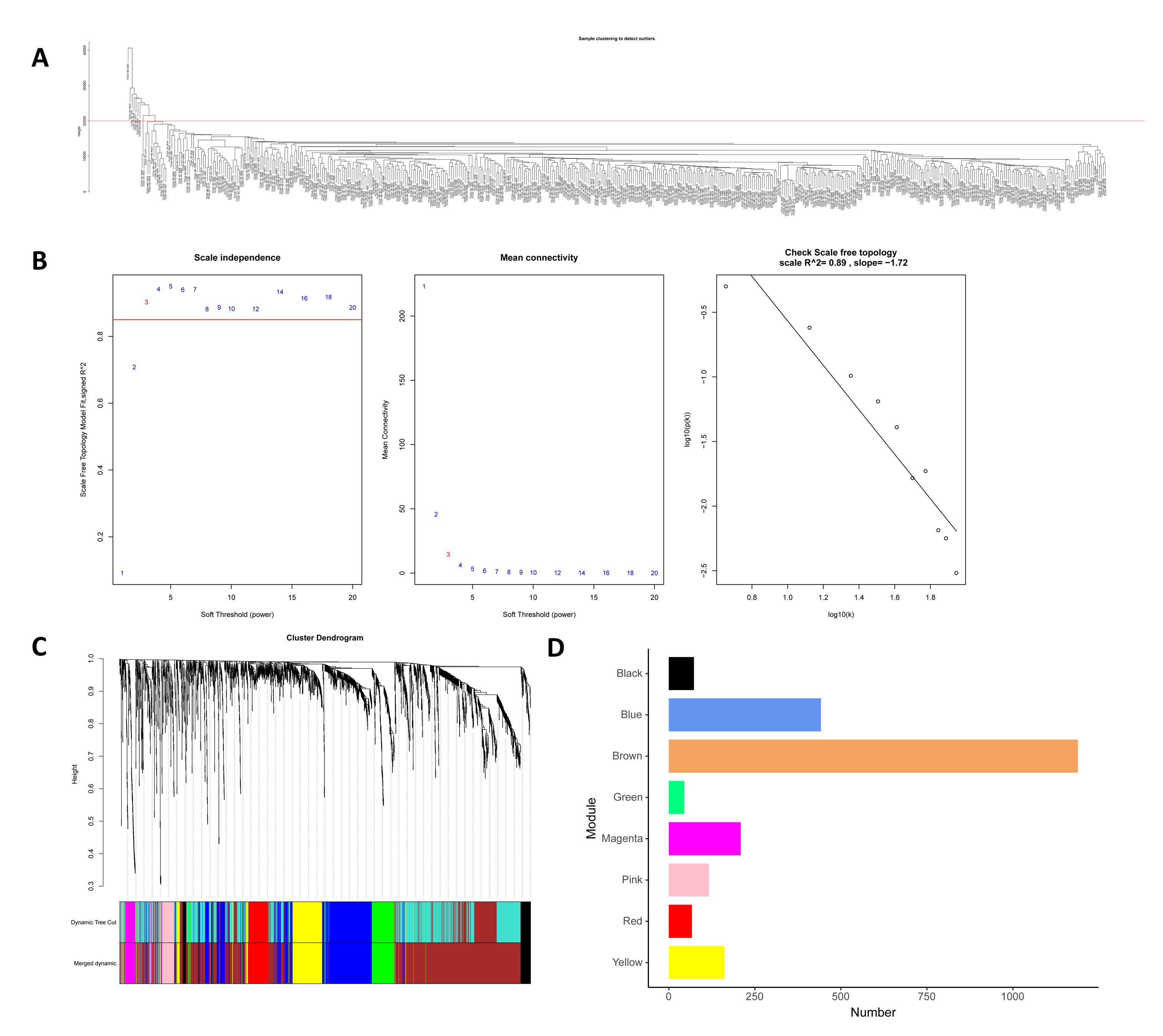

Supplement: Supplementary Figure 4 — The analysis of LUAD immune-gene co-expression networks. (A) A sample clustering. (B) LUAD immune-gene co-expression networks by setting four as a threshold for scale-free network. (C) Dendrogram of all differentially expressed genes. (D) Gene numbers in each module. [file Image_4.tif]
